# Treating Child Disruptive Behavior in High-Risk Families: A Comparative Effectiveness Trial from a Community-Based Implementation

**DOI:** 10.1007/s10826-015-0322-4

**Published:** 2015-11-26

**Authors:** Mariëlle E. Abrahamse, Marianne Junger, Mirjam A. M. M. van Wouwe, Frits Boer, Ramón J. L. Lindauer

**Affiliations:** De Bascule, Academic Center for Child and Adolescent Psychiatry, Amsterdam, Netherlands; Department of Child and Adolescent Psychiatry, Academic Medical Center, University of Amsterdam, Amsterdam, Netherlands; Faculty of Behavioural, Management and Social Sciences, University of Twente, Enschede, Netherlands

**Keywords:** Disruptive behavior, Parent management training program, Parent–child interaction, Community mental health

## Abstract

Parent management training programs have proven the most effective way to treat child behavior problems. This study reports on an effectiveness trial of a community-based implementation of Parent–Child Interaction Therapy (PCIT) in comparison with the Dutch-developed Family Creative Therapy (FCT). Forty-five children (58 % boys) aged between 32 and 102 months (*M* = 67.7, *SD* = 15.9) were referred for treatment, and they and their parent(s) were randomly assigned to PCIT or FCT. Treatment effectiveness was measured primarily by the degree of improvement on child behavior problems, using the Eyberg Child Behavior Inventory. Secondary outcomes included parent and teacher report data and independent observations of parenting skills and child behavior. During the trial, randomization was violated by treatment crossovers (from FCT to PCIT). Intention-to-treat analyzes revealed no significant differences in the primary outcome at 6-month follow-up, but interpretation was hampered by the crossovers. Subsequent treatment-received analyzes revealed significant interaction effects between time and treatment condition, with greater improvements in child behavior and parenting skills for PCIT families compared to FCT families. Analyzes on families that fully completed the PCIT protocol also showed higher treatment maintenance at follow-up. The treatment-received analyzes indicated promising results for the effectiveness of PCIT in treating young children’s disruptive behavior problems in a high-risk population. However, caution in generalizing the conclusions is needed in view of the design difficulties in this study. Suggestions are made for enhancing treatment delivery in daily practice, and clinical implications are noted.

## Introduction

Disruptive behavior disorders are highly prevalent among young children (Lavigne et al. 2009) and have been identified as the most common reason for referral to mental health services in that population (Loeber et al. 2000). Research in recent decades has revealed strong associations between these childhood adversities and developmental problems later in life in several domains (Frick and Nigg 2012; Tremblay 2000). Without effective treatment, the disorders have a high degree of persistence and can worsen over time (Bongers et al. 2004; Tremblay 2006).

Long-term outcomes include academic difficulties in late school years (McGee et al. 2002), unemployment, family problems (Maughan and Rutter 2001), and mental health problems such as depression, anxiety disorders, addiction, and antisocial personality disorders (Oldehinkel and Ormel 2014). An early diagnosis of a disruptive behavior disorder is also a serious risk factor for subsequent youth offending, adult crime, and interpersonal violent behavior, including anti-social behavior and substance abuse (McCord et al. 2001). Such negative outcomes result in higher costs for educational, mental health, law enforcement, and social services—estimated at ten times higher for children with disruptive behavior disorders than for children without problems (Lee et al. 2012; Scott et al. 2001). Given the high prevalence and persistence of serious behavioral problems and the costly trajectories of the children involved, this population is now a source of considerable public health concern. To reduce the risks of negative developmental outcomes and high public costs, early intervention is essential for young children with disruptive behavior problems.

Parent management training (PMT) programs, which target parents as the primary agents of change, have been found to be the most effective strategy to turn children with disruptive behavior away from disadvantaged trajectories (Eyberg et al. 2008; Weisz and Kazdin 2010). The accumulating empirical support for manualized PMT programs has resulted in their rapid worldwide dissemination in recent years. There is also increasing interest in the applicability of PMT programs in clinical practice under real-world conditions (Gardner et al. 2010). However, delivery of PMT programs (or evidence-based interventions in general) under real-world conditions is complex, and concerns have been raised about how compatible such interventions might be with everyday clinical practice (Weisz et al. 2015).

A review of youth psychotherapy outcome research (Weisz et al. 2005) has tested the clinical representativeness of studies in terms of three criteria: (1) study enrollment, (2) treatment providers, and (3) settings where treatment took place. It was found that most studies took place in settings created for research (e.g., university clinics) and included young people who were recruited rather than clinic-referred or treatment-seeking (Weisz et al. 2014). Treatment was often delivered not by clinical practitioners but by graduate students or other individuals dependent on the researcher for their employment. Although there is a growing need to test PMT programs in everyday clinical practice, previous research has identified a number of problematic factors. First, there are concerns about the treatment fidelity of practitioners, who may adapt interventions because they consider the protocol unsuitable for more complex cases (Michelson et al. 2013). Second, conducting more comprehensive studies such as randomized controlled trials (RCTs) is challenging in clinical practice, given the multiple aspects of variation and the difficulties in achieving standardization (Craig et al. 2008). Third, the engagement of parents and children in treatment and research presents a challenge to treatment effectiveness in real-world community mental health settings. High-risk populations (including families with low socioeconomic status or minority ethnic backgrounds) are overrepresented in child welfare services, but they remain understudied populations. Studies focusing on these groups have shown high attrition, which compromises treatment effectiveness (Fernandez and Eyberg 2009; Reyno and McGrath 2006). A fourth problem is that effect sizes in PMT programs remain small to moderate (Piquero et al. 2009; Weisz and Kazdin 2010).

Parent–Child Interaction Therapy (PCIT; Zisser and Eyberg 2010) is a well-established, US-developed PMT program for children aged 2–8 who have disruptive behavior problems. PCIT teaches authoritative parenting, including nurturance, good communication, and firm control, in two stages of therapy focused on changing dysfunctional parent–child interactions. PCIT has been disseminated to Australia, Puerto Rico, and several European and Asian countries (McNeil and Hembree-Kigin 2010), and its effectiveness in improving parent and child behavior after treatment has been widely supported in studies in different cultures (e.g., Leung et al. 2015; McCabe et al. 2012; Thomas and Zimmer-Gembeck 2007). Post-treatment maintenance of PCIT outcomes has also been demonstrated (Eyberg et al. 2014), and evidence for its usefulness in real-world settings is increasing (e.g., Lanier et al. 2014; Lyon and Budd 2010; Pearl et al. 2012). Although PCIT was originally developed to treat child disruptive behavior disorders, it has since been employed successfully in other populations, including children in foster care (Mersky et al. 2014), children with developmental delays (Bagner and Eyberg 2007), and children with autism spectrum disorders (Ginn et al. 2015). Over the past decade PCIT has also been successfully adapted to serve the needs of high-risk families in the treatment and prevention of child maltreatment (e.g., Chaffin et al. 2004, 2011; Kennedy et al. 2014; Thomas and Zimmer-Gembeck 2011, 2012).

Although PCIT is well researched internationally, European research on its effectiveness is still limited. A pilot study without a clinical control group has shown promising results (Abrahamse et al. 2012), but further testing is needed in more comprehensive research designs. Research studies in real-world clinical settings could contribute to the international evidence on PCIT. Previous research on another PMT program from the US known as Incredible Years, adapted for use in the Netherlands, found effect sizes in the Dutch context similar to those in the country of origin (Gardner et al. 2015; Posthumus et al. 2012). Other Dutch outcome research on Incredible Years within socioeconomically disadvantaged ethnic minority populations has also shown that parents and children with disruptive behavior problems in those groups could benefit from a PMT program (Leijten et al. 2015). Furthermore, the Western cultural concepts seem similar for the Dutch parents relative to parents in the US. For example, the authoritative parenting style including autonomy-oriented behavior and emotional warmth was commonly found in Dutch parenting (Van der Bruggen et al. 2010). Because PCIT teaches parents to use authoritative parenting, Dutch parents may react similarly to treatment.

Family Creative Therapy (FCT, a literal translation of the Dutch Gezins-Creatieve Therapie) (Beelen 2003; Smits 2002) is a frequently used, Dutch-developed form of art psychotherapy. It is available in most Dutch community mental health services and is commonly provided in clinical practice for malfunctioning interaction patterns in families with children aged 2–16. A number of theoretical frameworks underlie FCT, including systemic therapy approaches (Minuchin 1974; Satir et al. 1994; Van der Pas 2009) and learning by experience (Kolb 1976). It also draws on positive psychology, focusing on a positive goal rather than a problem (Conoley and Conoley 2009; Smits 2008). FCT is used to improve communication between family members in families with maladaptive parent–child interactions and/or parenting difficulties (including high-risk families or families with children with learning impairments). FCT is contraindicated for parents who have substance use problems or are currently involved in major family incidents such as divorce. Empirical evidence supporting the effects of FCT, as well as international literature, is lacking. No controlled research design or standardized outcome measures have yet been employed. There is no lack of detailed case reports, however (e.g., Witte 2013), that describe improvements in family interactions and functioning, often maintained at follow-up assessments 2–5 years later.

Unlike some PMT programs, both PCIT and FCT engage the parent(s) and the child. In FCT, all siblings are involved, as treatment focuses on family interaction as a whole. Both interventions aim to improve parent–child interactions; they create opportunities for parents to practice new skills during sessions—a treatment component strongly associated with program effectiveness (Kaminski et al. 2008). Although there are similarities between PCIT and FCT, their delivery also differs. While PCIT focuses mainly on the verbal aspects of parent–child interaction and on child compliance, FCT additionally emphasizes non-verbal interaction and cooperation. PCIT is characterized by a structured treatment protocol, whereas the FCT protocol requires more parental input in formulating specific treatment goals. The goals in PCIT focus mostly on reducing the child’s disruptive behavior, while the FCT treatment goals are formulated positively and usually focus on improving communication between family members, such as giving more positive attention to siblings without disruptive behavior problems.

In sum, Dutch research on the effectiveness of PCIT and FCT is limited, and more research is needed to gain or improve empirical support for these interventions, particularly in real-world clinical practice. The present study assesses the effectiveness of PCIT in families with children with disruptive behavior problems in a RCT conducted in a community mental health setting. Specifically, we address the following research questions: (1) What are the effects of PCIT in comparison with FCT in reducing children’s disruptive behavior problems? (2) What are the effects of PCIT and FCT on other, related child and parent outcomes?

## Method

### Participants

Children (aged 2–8 years) were referred to an academic center for child and adolescent psychiatry, which operates a large community mental health service for children, adolescents, and families with psychiatric problems in Amsterdam. The funding of care and services in the community mental health center comes from the local government and the Dutch health insurance system. All families had sought treatment and had been referred through the usual community channels. Recruitment for study participation took place from June 2009, to December 2012. Data collection including follow-up continued until May 2014. Children could be included in the study if (1) disruptive behavior problems were a reason for their referral, (2) they were aged between 2 and 8, (3) their parents were Dutch- or English-speaking. Child exclusion criteria were clinical signs of developmental or physical disabilities (e.g., learning impairments, deafness), but no children with such disabilities were referred to our department during the recruitment period. Family exclusion criteria were parental learning disabilities (IQ < 80), parental substance use disorders, and serious concerns about a child’s safety in the home situation, with a high risk of out-of-home placement; no families were excluded on those risk factors during the selection stage.

**Fig. 1:**
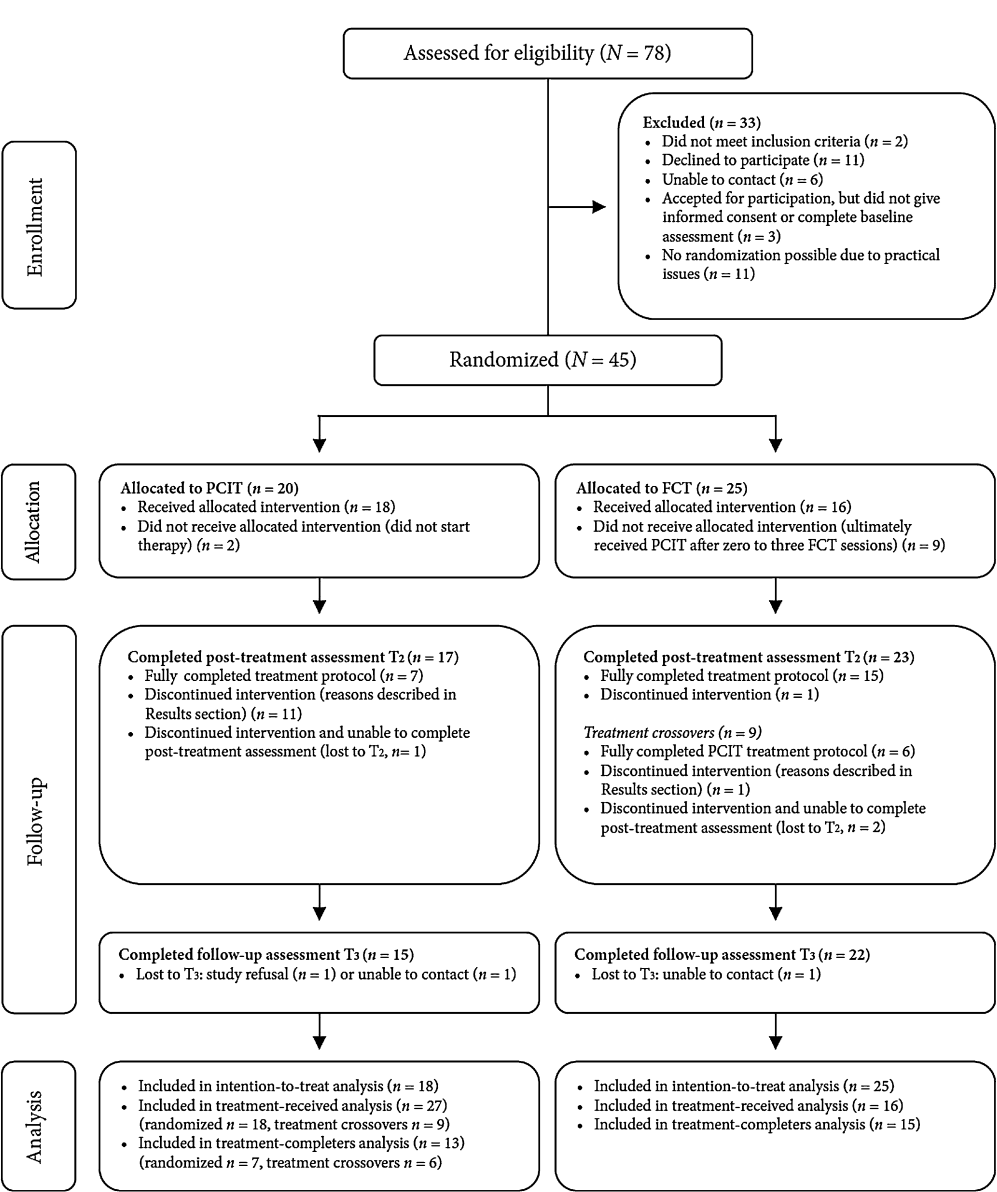
CONSORT flow diagram: Parent–Child Interaction Therapy (PCIT) and Family Creative Therapy (FCT)

Of the participating children (*N* = 45), the largest group (42.2 %) were referred by another child mental health service. Twelve families (26.7 %) were referred by child protection services, eight families (17.8 %) were internal referrals from other departments of the community mental health center, and six families (13.3 %) were referred by a general practitioner. After informed consent, families were initially assigned to PCIT (*n* = 20) or FCT (*n* = 25) using an allocation ratio of 1:1, including block randomization stratified by child age and gender (Fig. 1). Two families allocated to PCIT did not begin therapy; one of these moved to another city after inclusion, and in the other family significant signs of sexual abuse emerged. Sexual abuse is not typically a contraindication for PCIT, unless the parent participating in treatment is thought to be the perpetrator, which was the case in this family. Nine families initially allocated to FCT were transferred to PCIT after zero to three FCT sessions. In six of those cases, the parents or the referring counselor disagreed with the randomization outcome. The other three families crossed over after clinical judgment by the family creative therapist; in two such cases, working with constructive materials seemed inappropriate given the severity of the child’s behavior problems; the other child was very young, 32 months, and had trauma symptoms, so that the therapist deemed a play-based therapy like PCIT more suitable.

For the purpose of the intention-to-treat (ITT) analysis, the baseline characteristics of all families initially allocated to PCIT or FCT are summarized in Table 1 for the total sample and the two treatment conditions. No differences were found between treatment conditions (Chi square tests or *t* tests, *p* < .05), except in family income. Child age ranged from 32 to 102 months. One child met the inclusion criteria at referral, but was 8.5 years of age by the time of the baseline assessment; we decided not to exclude that family. The biological mothers of all the participating children were involved in the treatment, and the biological fathers of 20 children (46.5 %) were also actively involved.

**Table 1:** Demographic information for the total sample and by randomization group

	Means (SD) or percentages			
	Total (*N* = 43)	PCIT (*n* = 20)	FCT (*n* = 25)	*p*
*Child characteristics*				
Age (months)	67.7 (15.9)	69.8 (11.7)	66.1 (18.8)	.449
Gender (% male)	57.8	60.0	56.0	.787
Race (% Caucasian)	69.8	60.0	76.0	.226
Maltreatment history (% reported in client file)	71.1	75.0	68.0	.607
No diagnosis for disruptive behavior (%)	33.3	35.0	32.0	.931
ADHD diagnosis (% significant symptoms)	52.4	42.1	60.9	.226
ODD diagnosis (% significant symptoms)	39.0	38.9	39.1	.987
CD diagnosis (% significant symptoms)	12.5	26.3	4.3	.075
*Family characteristics*				
Mother’s age (years)	35.7 (5.6)	37.3 (5.5)	34.5 (5.5)	.095
Family status (% single-parent)	40.0	45.0	36.0	.540
Family income (% <€1000 per month)	15.2	25.0	9.1	.046*

ADHD symptoms include both inattentive and hyperactive behavior

* *p* < .05

### Procedure

Referred families meeting the inclusion criteria received information about the purposes and procedures of the study. After parents provided their written informed consent, they were individually randomized to PCIT or FCT. The randomization list was prepared by a methodologist and managed by a researcher who had no further involvement in the study. After randomization, that researcher communicated the assigned treatment condition directly to the coordinating therapist, who was responsible for matching an available therapist to the family. This procedure was established to maintain the blindness of the research team members. Baseline assessment (T_1_) was conducted prior to the start of the intervention, and post-treatment assessment (T_2_) was carried out immediately after the researcher was informed about treatment completion or termination. Follow-up assessments (T_3_) were performed 6 months after the post-treatment assessment. Additionally to the parent reports, each child’s teacher was asked to complete some questionnaires at the time of the baseline and follow-up assessments. The study received approval from the Medical Ethics Committee of the Academic Medical Center of Amsterdam and was registered in the Dutch trial register (ID: NTR1743).

### Treatment Conditions

#### Parent–Child Interaction Therapy

Parents and children allocated to PCIT received an intervention that progressed through two distinct phases: Child-Directed Interaction (CDI) and Parent-Directed Interaction (PDI) (Zisser and Eyberg 2010). Both phases started with a parental didactic session followed by weekly coaching sessions of approximately 1 h. The therapist coached the parents in vivo through a one-way mirror and a wireless headset. Alongside the treatment sessions, parents were given homework sheets to record their daily skill practice at home during special playtime with their child. In the CDI phase, the parents were taught to follow the child’s lead during play and were coached to use praise, reflection, imitation, description, and enthusiasm/enjoyment (PRIDE skills). This phase of treatment was intended to enhance the parent–child relationship. The number of sessions was dependent on the parent’s mastery of the skills (10 behavioral descriptions, 10 reflections, 10 labeled praises, and fewer than three commands, questions, or negative verbalizations during a 5-min observation). Once the parents met these mastery criteria, they proceeded to the PDI phase of the treatment, designed to improve child compliance. Parents were taught and coached to provide clear commands and to use consequences for compliance (praise) and non-compliance (timeout). Treatment ended when parents reached mastery criteria for PDI, as described in the original treatment protocol, and had rated their child’s behavior as well within normal limits (Eyberg Child Behavior Inventory Intensity Scale ≤114). Although PCIT is manualized, the intervention was not time-limited. Each family received the number of sessions necessary for the parents to master the CDI and PDI skills, in order to reduce their child’s disruptive behavior below clinical levels.

#### Family Creative Therapy

Families allocated to FCT (Beelen 2003) were expected to bring all siblings into treatment. FCT consisted of 10 sessions of approximately 1 h every 2 weeks, with a possible extension to as many as 15 sessions. Parents also received homework assignments to help them effectively use the time between sessions. In comparison to PCIT, the focus of FCT was more on the parents, on creating ‘good-parent’ experiences and improving their parental competence. The key feature was the opportunity for the parents to discuss each session’s program and strategy beforehand with the therapist and to evaluate the sessions afterwards. Parents were co-responsible for the content, procedure, and role-taking during the family sessions. The premise was that carefully prepared creative work (e.g., a mosaic mirror or a diorama) offered an opportunity for parents to practice childrearing skills, such as leading the children while taking into account their individual capacities, regulating amounts of attention, setting limits, and regulating the children’s emotions—all with the aim of creating experiences of success for all family members.

During the initial FCT sessions, parents were asked to formulate their goals for the therapy. Subsequently, the therapist chose a creative task to suit the parents’ goals and capabilities, which could be successfully carried out during the family sessions. Prior to the session itself, the therapist prepared the parents while the children were with the co-therapist, and afterwards there was a separate parental debriefing. During the therapy sessions, and while all family members were working on the task, the therapist observed, consulted perhaps briefly with a parent, or gave extra support. The emphasis was on success in moving toward the goals in the domains the parents had formulated for themselves. FCT develops in six phases as a whole: motivation, activation, stimulation, practicing skills, insight, and a final stabilization phase.

#### Training and Treatment Integrity

Both PCIT and FCT had established procedures to monitor program fidelity. All therapists completed the formal training workshops and received additional supervision from the master trainers (PCIT) or the program developers (FCT). The training and supervision levels were similar for both interventions. In regard to the clinical representativeness, all PCIT and FCT therapists were practicing clinicians within the community mental health center, and not graduate students or researchers. Besides delivering PCIT or FCT, these therapists had diverse caseloads with broad arrays of problems. Consistent with the Dutch and international requirements for the PCIT and FCT training workshops, all therapists had completed higher education and had bachelor and masters degrees in mental health fields.

In accordance with the established protocols, all therapy sessions were videotaped. Unfortunately, due to practical problems (e.g., lost videotapes or problems with recording systems), videos were available for only 72 % of the participating families. Because therapists received additional supervision, one random treatment session for each family was coded for treatment integrity. Independent undergraduate or graduate research assistants coded the videos using component checklists for the specific treatment session in question. For PCIT, the fidelity checklists from the original treatment protocol were used. For FCT, component checklists were created on the basis of the treatment protocol and were approved by the program developers. For both types of intervention, treatment integrity was >70 % (72 % for PCIT and 78 % for FCT). Due to practical issues (e.g., lost videotapes or unavailable coders), only three quarters (74 %) of the videos could be double-coded by a second research assistant; the result was a high interrater reliability of .87 (intraclass correlation).

### Measures

The primary outcome was the level of child behavior problems, measured using the Intensity Scale of the Eyberg Child Behavior Inventory (ECBI; Eyberg and Pincus 1999). In the present study, we generally used the mother reports in our analyses, because those were available for all children and the number of paternal reports was smaller. However, since 46.5 % of the fathers were engaged in the treatment and fathers’ participation in PMT programs is considered important (Bagner and Eyberg 2003), we also included the available father reports in our analyses. Most of the assessment instruments we chose were commonly used measures in PCIT outcome research. In addition to the standardized questionnaires, parents completed a demographic questionnaire.

#### Eyberg Child Behavior Inventory

The ECBI is a widely used 36-item parent-report measure of disruptive child behavior. Specific behavior is rated on two scales: the Intensity Scale and the Problem Scale. The Intensity Scale measures the frequency of the child’s behavior along a 7-point scale (1 = never to 7 = always), and the Problem Scale measures whether the parent perceives the specific behavior as a problem. Good reliability and validity have been demonstrated both for the English version (Funderburk et al. 2003) and for the Dutch translation (Abrahamse et al. 2015). In the present study, the internal consistencies (Cronbach’s alpha) for the ECBI Intensity Scale were .93 for the mother reports and .95 for the father reports. The ECBI Problem Scale internal consistencies were .91 and .90 for mother and father reports respectively.

Additionally, teachers completed the adapted version of the ECBI relevant for school situations, the Sutter-Eyberg Student Behavior Inventory-Revised (SESBI-R; Eyberg and Pincus 1999). This 38-item questionnaire uses the same scoring and scale format as the ECBI (Intensity and Problem Scales) and it has good reliability and validity (Funderburk et al. 2003; Kirkhaug et al. 2012). The Cronbach’s alphas for the SESBI-R in the current study were .97 for the Intensity Scale and .96 for the Problem Scale. Both Dutch versions of the ECBI and SESBI-R were back-translated and approved by the publisher (Psychological Assessment Resources, PAR). According to the professional manual (Eyberg and Pincus 1999), the published cut-off scores were ≤132 (ECBI) and ≤151 (SESBI-R) for the Intensity Scale, and they were ≤15 (ECBI) and ≤19 (SESBI-R) for the Problem Scale.

#### Anxiety Disorders Interview Schedule

At the baseline assessment, the parent version of the Anxiety Disorders Interview Schedule (ADIS; Silverman and Albano 1996) was used to assess clinically significant levels of externalizing disorders in children, including attention-deficit hyperactivity disorder (ADHD), oppositional defiant disorder (ODD), and conduct disorder (CD). The ADIS is a semi-structured interview, and diagnoses are based on information about symptoms and their interference in daily life. Although the primary focus of the ADIS is on anxiety, the interview also assesses other related disorders such as mood and externalizing disorders. The ADIS interview was chosen above other assessment tools because it was a commonly used interview in our department and training on its administration was available. Only the questions for the externalizing disorders were used in the current study. Trained researchers (first and third authors) administered the ADIS, but no interrater reliability was assessed. However, the ADIS has been found to have good-to-excellent test–retest and interrater reliability (Silverman and Albano 1996).

#### Maltreatment Classification System

The Maltreatment Classification System (MCS; Barnett et al. 1993) was used to code whether children, on the basis of their records at referral, had been exposed to any subtype of maltreatment, including physical abuse, sexual abuse, emotional maltreatment, physical neglect of basic needs, and physical neglect by lack of supervision. Subtypes were coded on a 3-point scale (0 = *not reported*, 1 = *suspicions* or 2 = *reported*). Maltreatment was recorded only if there were one or more scores of 2 (reported). Two researchers scored the client records independently. In the event of disagreement, the most accurate classification was determined in consultation with a third researcher. The average agreement between observers (Cohen’s kappa) for the five MCS subtypes was .63.

#### Child Behavior Checklist

The Child Behavior Checklist (CBCL; Achenbach and Rescorla 2000, 2001) contains two broadband scales that are widely used to assess internalizing and externalizing behavior problems. Our study employed two versions: the CBCL for ages 1.5–5 with 100 items and the CBCL for ages 6–18 with 113 items. Mothers rated the items on a 3-point scale (0 = *not true,* 1 = *somewhat or sometimes true*, 2 = *very true or often true*). The Cronbach’s alphas in the present study were .85 for the Internalizing Scale and .93 for the Externalizing Scale.

Teachers completed the Teacher Report Form (TRF) for ages 1.5–5 and 6–18 (TRF; Achenbach and Rescorla 2000, 2001), measuring the child’s school functioning and behavioral problems in the same format as the CBCL. Internal consistencies were .79 for the Internalizing Scale and .93 for the Externalizing Scale. Good psychometric properties have been demonstrated for the Dutch versions of the CBCL and the TRF (Verhulst et al. 1996, 1997). To combine the CBCL and TRF age versions in the data analysis as single outcome variables, we calculated *T*-scores on the basis of the professional manual, with *T* ≥ 60 indicating clinical problem behavior.

#### Parenting Stress Index Short Form

The Dutch translation and adaptation of the reliable and valid Parenting Stress Index Short Form (PSI-SF; Abidin 1995) was used to measure parenting stress (De Brock et al. 1992). All 25 items were rated on a 6-point scale ranging from 1 (*completely disagree*) to 6 (*completely agree*). Reliability and validity for the Dutch version have been described as satisfactory (De Brock et al. 1992). In the present study, the sum of all items was used as an overall parenting stress scale, with internal consistencies measuring .95 for the mother reports and .97 for the father reports. According to published norms (De Brock et al. 1992), a sum score above 74 indicates a clinical level of parenting stress.

#### Therapy Attitude Inventory

At the post-treatment assessment, mothers were asked to complete the Therapy Attitude Inventory (TAI; Eyberg 1992), a 10-item consumer satisfaction measure addressing the impact of parent training on 5-point Likert scales, which vary depending on the specific item, but with higher scores indicating greater satisfaction. Items explore the parent’s perceptions and confidence with respect to the discipline techniques learned, the quality of the parent–child interaction, changes in the child’s behavior, and overall family adjustment. Sample items include “Regarding my confidence in my ability to discipline my child, I feel…,” with response options ranging from (1) *much less confident* to (5) *much more confident*, and “I feel the type of program that was used to help me improve the behaviors of my child was…,” with response options ranging from (1) *very poor* to (5) *very good*. Although there was no information about the reliability and validity of the Dutch translation, psychometric evaluation of the original version has demonstrated adequate reliability and validity (Brestan et al. 1999). The internal consistency of the TAI was .89 in the current study.

#### Dyadic Parent–Child Interaction Coding System

The Dyadic Parent–Child Interaction Coding System (DPICS; Eyberg et al. 2013) assesses the quality of parent–child interaction during three 5-min structured situations—Child-Led Play (CLP), Parent-Led Play (PLP), and Clean-Up (CU)—which require a cumulative degree of parental control. All our DPICS observations were conducted with the mother and the child. The child’s and the parent’s verbal and nonverbal behavior were observed and frequencies were counted by independent coders. For the present study, the categories were chosen that were most relevant to treatment outcome. Six composite categories were used, derived from the professional research manual (Eyberg et al. 2013, p. 161). The two child categories were Inappropriate Behavior (including Negative Talk, Negative Touch, Yell, and Whine, coded in all three situations); and percentage of Compliance (coded in PLP and CU only). The four parent categories were the percentage of Positive Following (coded in CLP only and including Behavior Descriptions, Reflections, Labeled Praises, and Unlabeled Praises divided by the total of parent verbalizations); the percentage of Negative Leading (coded in CLP only and including Commands, Questions, and Negative Talk divided by the total of parent verbalizations); Praise (the sum of all praises in the three situations, including Labeled and Unlabeled Praises); and Demandingness (the sum total of Indirect and Direct Commands, coded in all three situations). The independent coders were trained to 80 % agreement with the first and third authors. All observations were transcribed to monitor interrater reliability. In every video observation, a minimum of one random situation (CLP, PLP, or CU) was coded twice to estimate reliability. High interrater reliability (intraclass correlations) was established, ranging between .67 (Direct Commands) and .96 (Questions) for the parent categories and .68 (Yell) and .91 (Negative Talk) for the child categories.

### Statistical Analyses

All analyses were performed in SPSS, version 19. First, ITT analyses based on the initial randomization were performed on the primary outcome measure. These analyses included all participating families (*N* = 45), whether or not all assessments had been completed and regardless of which intervention they had actually received. Missing values were replaced according to the principles of the last-observation-carried-forward (LOCF) method. Because post-treatment and follow-up assessments were also carried out for most families that did not complete the treatment protocol, missing data was limited (see Fig. 1). Independent *t*-tests were used to examine pre-treatment differences. An ANCOVA was then performed to examine the post-treatment and follow-up differences between the two treatment conditions on the primary outcome measure (the ECBI Intensity Scale), with the baseline means entered as covariates.

To analyze group differences in outcome between the interventions that the families actually received, we subsequently performed treatment-received analyses on the final distribution (PCIT *n* = 27; FCT *n* = 16). On this treatment-received subsample, we conducted linear mixed models analyses to investigate whether both treatments led to significant improvements in primary and secondary outcomes over time and whether significant differences in effectiveness emerged between PCIT and FCT. All observations from every treatment participant were used, irrespective of missing data. Assessment times, treatment conditions, and the time × treatment condition interaction terms were entered into the model. Analyses were performed using an unstructured covariance matrix, as that model showed the best fit based on the smallest −2 log likelihood value (Twisk 2013).

Additionally, effect sizes were calculated by dividing the baseline and follow-up means by the pooled standard deviations, whereby 0.2 indicated a small effect, 0.5 a medium effect, and 0.8 or higher a large effect (Cohen 1992). A number of families did not fully complete the PCIT and FCT treatment protocols. In order to examine the consequences of the attrition for the outcomes regarding treatment effectiveness at post-treatment and maintenance at follow-up, we repeated the linear mixed models analyses on this treatment-completers subsample separately.

To determine whether the changes in child behavior were clinically relevant, we calculated clinical significance and reliable change indices (RCIs; Jacobson and Truax 1991) on the individual child level for the primary outcome measure, the ECBI Intensity Scale. Clinical significance at follow-up was established if the score had fallen below the published clinical cut-off score of 132. RCIs were determined by dividing the magnitude of change between baseline and follow-up scores on the Intensity Scale by the standard error of the difference score.

## Results

### Baseline Problem Levels

At the baseline assessment, a structured clinical interview, the ADIS (Silverman and Albano 1996), was administered to the mother to assess the presence of clinically significant levels of ADHD, ODD, and CD symptoms, based on diagnostic criteria from the Diagnostic and Statistical Manual of Mental Disorders, Fourth Edition (DSM-IV; American Psychiatric Association 2000). The ADIS was administered for 42 children. All children had been referred for disruptive behavior problems in the home or school setting, but for 15 of them (35.7 %) the mothers not reported clinically significant symptom levels meeting DSM-IV criteria for the various disorders. Eight children (19.0 %) met the criteria for ADHD only, three (7.1 %) for ODD only, and one (4.8 %) for CD only. Ten children (23.8 %) met the criteria for both ADHD and ODD, one child for ADHD and CD, and one child for ODD and CD. Three children met the criteria for all three disorders (ADHD, ODD, and CD). Chi square tests revealed no significant differences between the two treatment groups on the distribution of the diagnoses (Table 1).

Based on the criteria established by Barnett et al. (1993) for the MCS, 71.1 % of the children had been exposed to some subtype of child maltreatment, including physical abuse, sexual abuse, emotional maltreatment, physical neglect of basic needs, or physical neglect by lack of supervision. As noted above, signs of sexual abuse emerged in one family after its inclusion in the study, with the participating parent being the suspected perpetrator. Since sexual abuse is contraindicated for PCIT if the parent participant is the perpetrator, that family did not start treatment. The high prevalence of child maltreatment indicated that the study sample included a large proportion of high-risk families. Prevalence did not significantly differ between families allocated to PCIT and to FCT (Table 1).

Frequency analyses on maternal baseline data for the total sample revealed that the majority of the mothers reported elevated levels of parenting stress and child disruptive behavior. In more detail, 63 % of the mothers reported clinical levels of stress on the PSI-SF (*M* = 87.5, *SD* = 25.6). In terms of disruptive behavior problems, the majority of participating children were rated within the clinical range on the ECBI Intensity Scale (56 % of children, *M* = 142.7, *SD* = 32.3), the ECBI Problem Scale (61 %, *M* = 16.8, *SD* = 8.4), and the CBCL Externalizing Scale (75 %, *M* = 68.3, *SD* = 10.2). In addition, 65 % were rated within the clinical range for internalizing behavior problems (CBCL Internalizing Scale; *M* = 61.9, *SD* = 8.1).

For the teacher-reports, these means and percentages were lower. Nonetheless, the majority of the children were still reported by teachers to be within the clinical range on the TRF Externalizing Scale (62 % of children, *M* = 63.5, *SD* = 9.7), but not on the SESBI-R Intensity Scale (39 %; *M* = 130.5, *SD* = 49.3). Although elevated frequencies of child disruptive behavior were thus apparent in the school situation, most teachers did not perceive those behaviors as a problem. On the ECBI Problem Scale, 31 % of the scores were in the clinical range (*M* = 8.7, *SD* = 10.4). In comparison with the mother reports, clinical levels for internalizing behavior problems (TRF) were not frequently reported by the teachers (28 %, *M* = 56.9, *SD* = 7.8).

### Intention-to-Treat Analyses

All the families in the sample were first analyzed on the primary outcome measure, the ECBI Intensity Scale, on the basis of their initially allocated treatment condition (PCIT, *n* = 20; FCT, *n* = 25). The LOCF method was applied, whereby families were included regardless of whether they had completed all three assessments or crossed over to PCIT. The independent *t* test revealed no baseline difference on the ECBI Intensity Scale between the treatment conditions, *t*(43) = 0.608, *p* = .546. After adjustment for baseline means, no significant difference between the treatment conditions emerged on the ECBI Intensity Scale either at post-treatment, *F*(1, 42) = 2.17, *p* = .148, or at 6-month follow-up, *F*(1, 42) = 0.454, *p* = .504. Analyses omitting the LOCF method did result in one different primary outcome for the ITT analyses at post-treatment—with PCIT families showing marginally significantly lower post-test means than FCT families, *F*(1, 39) = 4.04, *p* = .051—but not at follow-up. Since family income levels significantly differed between groups, analyses were repeated with family income as a covariate, but all outcomes (LOCF and non-LOCF) remained unaffected.

### Treatment-Received Analyses

Because nine families had switched from FCT to PCIT treatment after randomization, we performed additional analyses to compare results on the primary and secondary outcome variables on the basis of the intervention *actually received* by the participating families. Unadjusted means and the results of the linear mixed models analyses assessing improvement over time and differences between treatment conditions are reported in Table 2. Independent *t* tests and Chi square tests revealed no significant differences between the two treatment-received groups on baseline means and demographics.

**Table 2:** Unadjusted means and within- and between-group comparisons in the treatment-received subsample

Measures	Group	*n*	Baseline (T_1_)		*n*	Post-test (T_2_)		*p* (*T*_1_ − *T*_2_)	*n*	Follow-up (T_3_)		*p* (*T*_2_ − *T*_3_)	*p* (*time* × *treatment*)	Effect size *d*	
			*M*	*SD*		*M*	*SD*			*M*	*SD*			Within-group T_1_ − T_3_	Between-group T_3_
*Child behavior*															
ECBI intensity (*mother*)	PCIT	27	144.6	31.1	26	103.7	36.4	<.001*	20	114.2	46.7	.205	.005*	0.77*	−0.50
	FCT	16	139.4	35.0	16	137.8	30.0	.857	13	133.2	25.8	.169		0.20	
ECBI intensity (*father*)	PCIT	14	156.5	22.8	12	101.9	37.6	.001*	10	116.6	43.9	.319	.024*	1.14*	−0.18
	FCT	8	132.3	40.3	7	133.7	36.6	.506	5	123.7	35.9	.984		0.22	
ECBI problem (*mother*)	PCIT	24	16.9	8.1	24	9.6	7.6	<.001*	17	9.1	10.2	.870	.250	0.84*	−0.32
	FCT	14	16.6	9.9	14	14.6	7.6	.460	13	12.0	7.5	.203		0.55	
ECBI problem (*father*)	PCIT	14	20.1	5.5	10	9.9	8.5	.001*	10	11.8	9.5	.675	.015*	1.08*	−0.43
	FCT	5	17.8	9.1	7	18.7	9.1	.109	4	16.3	11.4	.335		0.15	
CBCL internalizing (*T*-*score*)	PCIT	26	61.9	7.9	20	54.7	8.5	.002*	20	51.3	12.2	.164	.148	1.00*	−0.65
	FCT	14	61.9	8.9	16	59.4	7.6	.166	13	58.2	8.5	.862		0.44	
CBCL externalizing (*T*-*score*)	PCIT	26	68.0	11.1	20	62.7	10.0	<.001*	20	60.1	14.0	569	.224	0.63*	−0.42
	FCT	14	68.7	8.9	16	65.6	7.7	.156	13	64.7	7.3	.406		0.50*	
DPICS inappropriate behavior	PCIT	27	17.6	16.9	23	12.1	13.5	.126	20	9.4	9.0	.399	.986	0.61*	−0.39
	FCT	15	19.9	20.5	15	14.5	12.2	.452	11	13.2	10.3	.530		0.41	
DPICS % non-compliance	PCIT	27	45.3	23.8	23	33.2	25.9	.098	20	37.2	27.7	.397	.199	0.31	0.18
	FCT	15	32.8	25.1	15	40.6	25.3	.272	11	31.8	31.7	.631		0.03	
*Parenting stress*															
PSI-SF (*mother*)	PCIT	25	87.0	27.0	21	72.2	28.7	<.001*	18	71.1	33.6	.702	.520	0.52*	−0.32
	FCT	16	88.3	24.1	16	78.5	22.4	.231	12	79.7	16.8	.312		0.42	
PSI-SF (*father*)	PCIT	14	91.9	28.3	11	60.5	26.5	.002*	10	59.9	22.1	.838	.067	1.26*	−0.74
	FCT	8	85.3	28.2	7	86.4	30.6	.428	5	77.8	26.1	.270		0.27*	
*Parenting skills*															
DPICS % positive following	PCIT	27	7.3	4.4	24	15.1	11.4	.005*	20	18.2	15.8	.513	.128	−0.94*	0.86
	FCT	15	7.1	5.6	15	8.1	4.9	.570	12	8.0	5.7	.976		−0.16	
DPICS % negative leading	PCIT	27	43.0	12.1	24	26.9	11.0	<.001*	20	23.2	13.1	.222	.012*	1.57*	−1.51
	FCT	15	43.2	8.0	15	34.8	8.5	.010*	12	41.1	10.5	.108		0.48	
DPICS praise	PCIT	27	7.8	5.4	23	19.4	18.5	.006*	20	14.1	9.5	.132	.018*	0.81*	0.88
	FCT	15	8.3	6.7	15	5.3	4.0	.102	11	7.2	5.7	.786		0.17	
DPICS demandingness	PCIT	27	29.2	14.9	23	18.7	8.6	.003*	20	17.9	13.4	.474	.446	0.79*	−0.18
	FCT	15	28.4	18.8	15	25.0	12.7	.498	11	20.4	14.5	.354		0.48	

Tests of significance for assessment time and for time-treatment interaction used the baseline score as reference point. For DPICS % positive following and for DPICS praise, higher means indicate improvement. Asterisks in the T_1_ − T_3_ within-group effect size column indicate significant change from baseline to follow-up

* *p* < .05

Compared with the baseline scores, the mothers, fathers, and children who received PCIT showed significant improvements on all primary and secondary outcome measures at post-test and follow-up, with two exceptions: observed child inappropriate behavior showed significant change between baseline and follow-up, but not at post-test; and child non-compliance (DPICS) did not change significantly either at post-test or follow-up. For the families that received FCT, most outcome measures showed no significant improvements at post-treatment or follow-up. Negative parenting behavior (DPICS) did decline significantly after treatment, and that was maintained at follow-up. Child externalizing behavior (CBCL) decreased significantly between baseline and follow-up.

Some domains showed greater improvement after PCIT than after FCT, as revealed in significant interaction effects between time and treatment on the ECBI Intensity Scale (both parents), ECBI Problem Scale (father), DPICS Negative Parental Leading, and DPICS Praise. Within-group effect sizes (T_1_ − T_3_) were calculated, and for FCT these indicated low-to-medium effects ranging from 0.03 (Child Non-compliance) to 0.55 (ECBI Problem Scale), whereas for PCIT they indicated medium-to-high effects from 0.31 (Child Non-compliance) to 1.57 (Negative Leading). Between-group effect sizes at follow-up indicated low-to-medium effects for PCIT on child behavior (reported and observed) and parenting stress (PSI-SF), a high effect for PCIT on parenting behavior (DPICS), and a low effect for FCT on child compliance (DPICS).

Treatment satisfaction (TAI) was significantly higher among mothers who received PCIT (*M* = 39.9, *SD* = 7.3) than among those receiving FCT (*M* = 34.4, *SD* = 5.0), *t*(33.24) = 2.68, *p* = .011. On the teacher reports in both treatment conditions, no significant decrease was found between baseline and follow-up mean scores. Nor did significant between-group differences emerge in terms of baseline and follow-up difference scores for the SESBI Intensity Scale, *t*(26) = −0.17, *p* = .866, or the TRF Externalizing Scale, *t*(24) = −0.388, *p* = .701.

In regard to individual change, both clinical change and RCIs were calculated per case. For 40 % of the mothers who received PCIT, as well as a smaller proportion of the FCT mothers (15 %), a reliable and clinically significant change at follow-up was evident in the frequency of their child’s disruptive behavior (ECBI Intensity Scale). These mothers now rated their child’s behavior within the range of normal functioning (traditional clinically significant change), and a statistically reliable change in their child’s reported behavior was measured between baseline and follow-up.

### Treatment-Completers Analyses

Of the 27 families that received PCIT, 14 families (52 %) did not fully complete the treatment protocol. Seven families dropped out before attending 10 sessions; seven others attended 10 or more sessions but did not completely finish the protocol. Treatment completion was defined as completing the PCIT protocol by reaching the mastery criteria for CDI and PDI skills. After premature termination of PCIT, data collection for most families was continued. Of the 16 families that received FCT, just one family (6 %) dropped out before completing the 10 or 15 treatment sessions. For the entire study, the treatment attrition rate was 35 %.

There were several reasons why families terminated treatment before completing the protocol. Four families (27 %) left PCIT because parents felt treatment was no longer necessary. Three families (20 %) stopped showing up for treatment, and another three families (including the FCT dropout) had too many severe family problems to continue treatment. In five cases, parents did not actually drop out, but the therapist made a clinical judgment to end treatment before all completion criteria were met, due primarily to stagnation of therapeutic progress.

Families that fully completed the PCIT treatment protocol attended an average of 22 treatment sessions (*SD* = 8.0, MIN = 10, MAX = 39), with means of 11 CDI sessions (*SD* = 3.9) and 10 PDI sessions (*SD* = 4.0). The time-limited protocol of FCT included 10 sessions, but treatment for six families was extended to a maximum of 15 sessions. The FCT group as a whole received an average of 12 sessions (*SD* = 2.4). For the treatment completers, the total length of treatment differed significantly between the PCIT and the FCT participants, *t*(23) = 4.34, *p* < .001.

Table 3 shows the unadjusted means for the treatment-completers group. These reveal substantial post-treatment reductions in child behavior problems and parenting stress as well as considerable improvements in parenting skills. Significant interaction effects between time and treatment were found for the ECBI Intensity Scale (both parents), ECBI Problem Scale (father), CBCL Externalizing and Internalizing Scales, DPICS Child Non-Compliance, PSI-SF (father), and DPICS Positive Following, Negative Leading, and Praise. That indicates more improvement for PCIT than for FCT. Moreover, in the PCIT completers group a lower degree of remission was observed between post-treatment and follow-up, indicating higher treatment maintenance for families that fully completed the PCIT protocol in comparison with families that fully completed FCT. PCIT completers also showed higher effect sizes and higher treatment satisfaction (*M* = 45.4, *SD* = 3.6) than FCT completers (*M* = 34.0, *SD* = 4.93), *t*(23) = 6.25, *p* < .001. Because of the significant difference in numbers of sessions between PCIT and FCT, analyses were repeated to control for the number of sessions completed. Except for the DPICS Child Non-Compliance measure (*p* = .067), all interaction effects remained significant.

**Table 3:** Unadjusted means and within- and between-group comparisons in the treatment-completers subsample

Measures	Group	*n*	Baseline (T_1_)		*n*	Post-test (T_2_)		*p* (*T*_1_ − *T*_2_)	*n*	Follow-up (T_3_)		*p* (*T*_2_ − *T*_3_)	*p* (*time* × *treatment*)	Effect size *d*	
			*M*	*SD*		*M*	*SD*			*M*	*SD*			Within-group T_1_ − T_3_	Between-group T_3_
*Child behavior*															
ECBI intensity (*mother*)	PCIT	13	154.7	27.2	12	96.0	33.4	<.001*	11	103.0	43.1	.564	.002*	1.44*	−0.85
	FCT	15	139.5	36.2	15	137.5	31.1	.836	13	133.2	25.8	.190		0.20	
ECBI intensity (*father*)	PCIT	8	165.9	11.3	7	104.0	37.4	.017*	6	112.8	46.3	.564	.034*	1.57*	−0.26
	FCT	8	132.3	40.3	7	133.7	36.6	.506	5	123.7	35.9	.984		0.22	
ECBI problem (*mother*)	PCIT	11	19.9	6.8	12	10.3	8.7	.002*	11	9.7	10.9	.892	.349	1.12*	−0.24
	FCT	13	17.9	8.3	13	14.5	7.9	.230	13	12.0	7.5	.243		0.75	
ECBI problem (*father*)	PCIT	8	22.4	6.3	6	9.8	10.5	.013*	6	11.1	10.9	.854	.017*	1.47*	−0.46
	FCT	5	17.8	9.1	7	18.7	9.1	.109	4	16.3	11.4	.335		0.15	
CBCL internalizing (*T*-*score*)	PCIT	12	63.5	7.3	10	54.4	7.3	.002*	11	49.3	12.6	.169	.054*	1.38*	−0.83
	FCT	13	61.2	8.8	15	59.1	7.7	.247	13	58.2	8.5	.894		0.36	
CBCL externalizing (*T*-*score*)	PCIT	12	71.1	11.2	10	61.5	9.4	<.001*	11	56.1	13.9	.308	.009*	1.19*	−0.78
	FCT	13	68.5	9.2	15	65.2	7.8	.158	13	64.7	7.3	.474		0.46*	
DPICS inappropriate behavior	PCIT	13	18.6	19.7	13	9.9	13.0	.158	10	8.7	9.2	.739	.715	0.65	−0.46
	FCT	14	16.6	16.6	14	14.9	12.6	.797	11	13.2	10.3	.478		0.25	
DPICS % non-compliance	PCIT	13	49.9	25.1	13	28.2	22.8	.029*	10	30.6	25.7	.686	.044*	0.76	−0.04
	FCT	14	30.3	24.0	14	39.0	25.5	.272	11	31.8	31.7	.629		−0.05	
*Parenting stress*															
PSI-SF (*mother*)	PCIT	11	93.3	25.6	10	75.3	29.9	.014*	11	66.3	29.7	.747	.347	0.97*	−0.56
	FCT	15	89.7	24.3	15	79.1	23.0	.229	12	79.7	16.8	.315		0.48	
PSI-SF (*father*)	PCIT	8	107.5	18.6	6	63.3	27.8	.008*	6	53.2	15.4	.592	.007*	3.18*	−1.15
	FCT	8	85.3	28.2	7	86.4	30.6	.428	5	77.8	26.1	.270		0.27*	
*Parenting skills*															
DPICS % positive following	PCIT	13	7.6	4.1	13	18.4	12.0	.005*	10	22.4	13.3	.586	.005*	−1.51*	1.41
	FCT	14	7.3	5.7	14	7.2	3.6	.999	12	8.4	5.7	.680		−0.14	
DPICS % negative leading	PCIT	13	44.1	13.8	13	23.3	9.7	<.001*	10	21.1	16.7	.444	.014*	1.51*	−1.44
	FCT	14	43.3	8.3	14	35.2	8.7	.018*	12	41.1	10.5	.135		0.23	
DPICS praise	PCIT	13	8.5	4.0	13	23.3	21.4	.029*	10	16.0	10.1	.203	.022*	−0.99*	1.08
	FCT	14	7.8	6.7	14	4.7	3.3	.111	11	7.2	5.71	.502		0.10	
DPICS demandingness	PCIT	13	31.6	16.2	13	16.4	6.3	.007*	10	17.4	14.5	.816	.287	0.93*	−0.20
	FCT	14	27.1	18.8	14	23.3	11.3	.473	11	20.4	14.5	.524		0.40	

Tests of significance for assessment time and for time-treatment interaction used the baseline score as reference point. For DPICS % positive following and for DPICS praise, higher means indicate improvement. Asterisks in the T_1_ − T_3_ within-group effect size column indicate significant change from baseline to follow-up

* *p* < .05

Similar results emerged for individual change. In the PCIT treatment-completers group, higher percentages with clinically significant and with reliable changes were found. The majority of mothers at post-treatment (83 %) and follow-up (55 %) rated their child’s behavior within the range of normal functioning; reliable changes from baseline to post-treatment or follow-up were also apparent.

## Discussion

The aim of this study was to examine the effectiveness of the PMT programs PCIT and FCT in treating young children with disruptive behavior among high-risk families in the Netherlands. Our study satisfied the criteria for clinical representativeness put forward by Weisz et al. (2005) with respect to participant enrollment (community referrals), practicing clinicians as therapists, and a community mental health center as the treatment setting. As the importance of research for everyday clinical practice has been emphasized in recent years (Michelson et al. 2013; Weisz et al. 2015), our study helps to bridge the gap between science and practice. Most research on PCIT has used wait-list control conditions (e.g., Schuhmann et al. 1998; Thomas and Zimmer-Gembeck 2011) or adapted forms of PCIT (McCabe et al. 2012; Nixon et al. 2004) to compare treatment effects. The current study made a direct comparison between two different treatment approaches in two active conditions, a procedure not commonly seen in community-based implementation studies.

Multiple methods (using questionnaires, interviews, and observations) and multisource data collection procedures (including parents, independent observers, and teachers) were used to address the research questions. The randomization process suffered from some treatment crossovers, and the ITT analyses found no significant differences at follow-up between families that were initially allocated to PCIT or to FCT. Given the randomization violation, the ITT results were subject to limited interpretation, and it remains unknown whether an effect would have emerged without crossovers. As a consequence, we conducted additional analyses on the treatment-received and treatment-completers subsamples and regarded this study as a comparative effectiveness trial.

The results from the treatment-received and treatment-completers analyses suggested a preferred status for PCIT in the treatment of children with disruptive behavior problems and their parents. In comparison with FCT, parents who received PCIT reported significantly larger reductions in child disruptive behavior and were significantly more satisfied with the treatment. Mothers who received PCIT were also observed to interact with their children using more positive statements, including reflections, behavioral descriptions, and praises, and fewer negative leading statements, including questions, commands, and criticism. Significant decreases in parenting stress and in child internalizing problems were also reported among PCIT families. For all these outcome measures, the effects were maintained at the 6-month follow-up assessment. Parents who received FCT reported no significant improvements on any of these outcome measures, though we did observe a significant post-treatment decline in negative leading behavior and a significant follow-up decline in child externalizing behavior (CBCL) by FCT parents. Effect sizes and analyses examining individual change confirmed the preferred status of PCIT, with the majority of mothers who completed it reporting reliable change and rating their child’s behavior within the range of normal functioning. Despite the significant improvements in the PCIT families, however, a substantial percentage of the mothers still did not report reliable and clinical changes in their child’s behavior.

Surprisingly, beyond the increase in child compliance after PCIT completion, no significant changes were observed in children’s inappropriate verbal and non-verbal behavior in both treatment groups. The high variance between means at the baseline, post-treatment, and follow-up assessments may explain why changes were not large enough to be significant. Although child categories of the DPICS are not commonly reported in PCIT outcome studies, a recent study on discriminating families with ODD or CD children and families with children without a diagnosis using the DPICS, revealed no differences between these groups on child inappropriate behavior (Bjørseth et al. 2015). Therefore, we encourage including DPICS child behavior categories in future research, in order to study discrepancies between observed and reported child behavior. Also, it is important to investigate the sensitivity of the DPICS to observe actual child behavior and to detect change between baseline and post-treatment assessments.

Despite the fact that the subsample size of the fathers included in this study was small, results suggested that fathers who were actively involved in treatment did benefit from PCIT in similar ways to mothers in terms of diminishing child behavior problems and parenting stress. These findings were comparable to other PCIT outcome research that included fathers (Schuhmann et al. 1998). For FCT, however, fathers did not report significant improvements.

Although caution is required in the interpretation of our findings that PCIT was more effective than FCT, some ideas can be mentioned why PCIT was superior to FCT for children with disruptive behavior problems. For example, the theoretical model of PCIT may be closer to theoretical models about the etiology of disruptive behavior, such as the use of the social learning theory in attempt to reduce the coercive pattern in parent–child interactions (Patterson 1982). In addition, PCIT includes the technique of differential social attention, which may have contributed to the change in the child’s behavior (Zisser and Eyberg 2010). In comparison to FCT, PCIT also teaches parents to use time-out as a disciplinary technique and teaches them to respond consistently to their child’s behaviors. These program elements were associated with larger effect sizes in the reduction of child disruptive behavior and the improvement of parenting skills (Kaminski et al. 2008). Another possible explanation may be that PCIT was more intense with on average 22 weekly sessions compared to 12 bi-weekly FCT sessions.

Similarly to previous community-based PCIT studies (Lyon and Budd 2010; Pearl et al. 2012), the attrition rate for PCIT in our study was high (52 %). Also, this attrition rate for PCIT was higher than for the 10 to 15-session FCT (6 %). However, 50 % of families that did not complete the PCIT treatment protocol did take part in at least 10 sessions. Although findings from our study show that those families were able to benefit from PCIT treatment sessions without completing the full protocol, results also revealed a more substantial gain for families that achieved the specific mastery criteria of the CDI and PDI skills as prescribed for treatment completion. Higher treatment maintenance outcomes for treatment completers may indicate that families that make more improvement are also more likely to complete treatment, especially given that lack of improvement was a frequent reason for premature termination of PCIT. Such findings are also consistent with previous PCIT outcome research showing that dropouts had poorer long-term outcomes (Boggs et al. 2005). Terminating PCIT before reaching mastery criteria may constitute failure experiences in these families, which could in turn undermine the long-term effectiveness of treatment.

A previous study on PCIT that preceded the treatment proper with a motivational intervention to discourage attrition found higher program retention for referred families with limited motivation (Chaffin et al. 2009, 2011). Because some high-risk families do not receive treatment voluntarily, but are referred by child protection services, a motivational intervention might be useful to support such families in completing treatment. Also, a standard 12-session PCIT protocol has also been studied (Thomas and Zimmer-Gembeck 2012), with treatment outcomes that were either positive or significantly better than outcomes for the original non-time-limited PCIT protocol. This would also be a relevant direction for future research, as well as an implication for practice, in particular for families that are motivated but do not succeed in reaching mastery criteria. Similar to the higher treatment retention found for FCT families, the 12-session study underlined the benefits of a clear end-point—not only for parents, but also for policymakers and professionals in clinical practice, in view of the upcoming trend to provide shorter treatments in order to reduce the costs of services. Given the high attrition rates, especially in community mental health settings, future research is recommended on the additional motivational components and the restricted number of treatment sessions. That may inhibit dropout and improve the feasibility of PMT programs in everyday practice.

The present study included a large percentage (71 %) of children exposed to maltreatment. Although the study did not focus on preventing child maltreatment or improving parent–child interactions after maltreatment, evidence is growing on the effectiveness of PCIT in the prevention of child maltreatment (Thomas and Zimmer-Gembeck 2011). That is relevant because PCIT teaches parental skills that are effective, nonviolent alternatives to physical discipline. Moreover, in families where parents have been physically abusive, PCIT has been found effective in reducing future reports of physical abuse (Chaffin et al. 2011). However, another recent study on the prevention of child maltreatment in a community mental health setting did not find large effects for PCIT (Lanier et al. 2014). Given the high prevalence of maltreatment in the current study, and in the light of the previous literature, additional research on the prevention of child maltreatment in the Dutch context is advised.

Although PCIT parents reported significant more improvements in terms of child disruptive behavior problems compared to FCT parents, significant evidence reflecting such improvements was not apparent in the teacher reports for either the PCIT or the FCT children. Before the start of treatment, teachers had reported less clinical-range student behavior than mothers, suggesting low agreement between teachers and parents about children’s problem behavior. Discrepancies between mother and teacher ratings may reflect differences in the contexts where informants observe the behavior as well as differences in perceptions (De Los Reyes and Kazdin 2005). Several factors might explain the inconsistency in our findings. Parents and teachers may agree about which children have the severest problem behaviors, but parents may be more sensitive to those behaviors. The discrepancies between parent and teacher reports might have also been a consequence of the high comorbidity in our sample; behavior problems associated with ADHD tend to be less context-specific, while children may exhibit ODD problems in a single context, particularly if that context is not well structured. And because children moved on to other grades during the treatment phase, the teachers that completed the baseline questionnaires were usually not the same ones that completed the follow-up ones.

The overall findings of our study contribute to the literature on the transportability of parenting interventions across countries and cultures. Except the translation, PCIT did not require any substantial cultural adaptation to work effectively in a new environment. It produced similar changes on similar measures, consistently with the findings reported in the meta-analysis by Gardner et al. (2015). The current study could therefore provide an important impetus for the international dissemination of effective PMT programs in clinical practice. Nevertheless, some limitations of our study do need to be noted. We believe these relate to doing research in clinical practice outside a university clinic. First, although all children were referred for disruptive behavior problems, we did not screen the children for eligibility for inclusion. As a consequence, a large percentage (35 %) of the children in our sample did not have a clinically significant level of ADHD, ODD, or CD on the structured clinical interview (ADIS). Hence, one limitation may be that the study sample was smaller and more heterogeneous than samples from research clinics; on the other hand, our research is more reflective of real-world clinical practice. Second, for some families, disagreement with the randomization outcome arose, so that they ultimately received PCIT rather than the allocated FCT. That constituted a violation of the randomization principle in the controlled trial; it required additional analyses and therefore necessitates caution in generalizing our conclusions. A third issue is that our outcome measures were better suited to the PCIT treatment approach than to that of FCT. It therefore came as no surprise that greater improvements in parenting skills (DPICS) were seen in the PCIT group, since those were criteria that parents had to master to progress through that treatment. The primary focus of PCIT is to change the behavior of one child in the family. FCT focuses more on changing the interaction patterns in the family as a whole, leading to more enjoyment in parenting and more positive behavior. The outcome measures assessed child behavior and specific parenting behavior; they did not assess family interaction patterns. Accordingly, they were not suited to determining whether the aims of FCT were achieved. At the same time, beyond the fact that the ECBI and DPICS are both part of the PCIT intervention, it is important to point out that significant improvements among PCIT families were seen on additional outcome measures as well, including child internalizing behavior problems and parenting stress—improvements that were not seen in the FCT condition.

The comparative effectiveness trial reported on here gives modest support to the evidence base for PCIT as an intervention to treat child disruptive behavior problems in high-risk Dutch families. Our findings provide evidence for the successful international dissemination of this PMT program in real-world clinical practice. Although the challenges of randomization formed a limitation in interpreting the effect sizes of outcomes, the fact that we implemented the trial in a real-world context makes the findings promising from the standpoint of dissemination. Despite the study limitations, our results suggest that PCIT is preferable to FCT for treating young children with disruptive behavior problems. Replication in other samples and settings is needed before more definite conclusions can be drawn about the effectiveness of PCIT in the Netherlands.
